# Enzyme-activated intracellular drug delivery with tubule clay nanoformulation

**DOI:** 10.1038/srep10560

**Published:** 2015-05-15

**Authors:** Maria R. Dzamukova, Ekaterina A. Naumenko, Yuri M. Lvov, Rawil F. Fakhrullin

**Affiliations:** 1 Bionanotechnology Lab, Institute of Fundamental Medicine and Biology, Kazan Federal University, Kreml uramı 18, Kazan, Republic of Tatarstan, Russian Federation, 420008; 2 Institute for Micromanufacturing, Louisiana Tech University, 911 Hergot Ave., Ruston, LA, 71272, USA

## Abstract

Fabrication of stimuli-triggered drug delivery vehicle s is an important milestone in treating cancer. Here we demonstrate the selective anticancer drug delivery into human cells with biocompatible 50-nm diameter halloysite nanotube carriers. Physically-adsorbed dextrin end stoppers secure the intercellular release of brilliant green. Drug-loaded nanotubes penetrate through the cellular membranes and their uptake efficiency depends on the cells growth rate. Intercellular glycosyl hydrolases-mediated decomposition of the dextrin tube-end stoppers triggers the release of the lumen-loaded brilliant green, which allowed for preferable elimination of human lung carcinoma cells (А549) as compared with hepatoma cells (Hep3b). The enzyme-activated intracellular delivery of brilliant green using dextrin-coated halloysite nanotubes is a promising platform for anticancer treatment.

The targeted delivery of drugs directly into biological cells needs nanoscale design of the pharmaceutic vehicles. Nanoscopic particles are promising drug carriers due to their adjustable size, shape, porosity and surface properties^1^. The unquestioned potential of nanosized carriers as platforms for drugs transportation into the cells has stimulated the design of a variety of liposome and micellar systems^2^, polymeric conjugates^3^, porous silica^4^ and magnetic nanoparticles^5^, graphene oxide nanosheets^6^, supramolecular containers^7^ and mesoporous carbon nanospheres^1^. Nanodrug transportation trough the cellular membranes accomplished with the controlled release of the encapsulated cargo triggered by an external or internal impact is the ultimate goal for stimuli-responsive drug nanocarriers^8^.

To achieve these, nanocontainers loaded with drugs are provided with the additional surface coating responsive towards the triggering stimuli^9^. So far, either external (laser irradiation^10^) and internal (intercellular pH gradient^11^) factors or a combination of both^12^ have been employed to initiate the intercellular release from internalised drug nanocontainers. Typically, drug-loaded nanoparticles are coated with a responsive coating (i.e. pH-responsive silane layer), which restricts the release while the carriers are outside the cells^13^. Drugs entrapped within the self-assembled block copolymer nanoparticles were released inside the cells after photolysis induced by laser irradiation or carbon nanotube decapping^14^. Although these approaches appear to be effective *in vitro*, the use of external triggers, such as laser irradiation, is not always applicable for the treatment of internal organs, whereas pH-triggered release requires the introduction of synthetic polymers and may lead to the increased toxicity.

Despite the numerous reports describing on-command drug delivery, it appears that the pH-responsive^14^, electric field^15^ or light-activated^10^ carriers are too complicated for the real-life applications. The systems based on the activation of the drug release by intracellular enzymes are regarded as a promising alternative^16^. Particularly, hydrocarbon molecules covalently linked to silanes were utilised to control the glycoside hydrolase-triggered release of anticancer drug doxorubicin from mesoporous silica nanoparticles (MSN) and demonstrated *in vitro* the effective action against cancer cells^17^. MSN are used in fabrication of drug delivery carriers^13^, although their applications are limited by small pore size (2–3 nm) and potential *in vivo* toxicity^18^. Carbon nanotubes are utilised as the potent drug carriers since they can be easily internalised by mammalian cells^19^ and provide the room for drugs until they reach the target cell, while the open tube end gives an access to the inner volume^20^. However, carbon nanotubes are regarded as potentially toxic materials^21^, thus stimulating the quest for the alternative tubular carriers^22^.

Clay nanotubes have been suggested as versatile nanocarriers^23^, combining the effective drug loading into tubule lumen and well-developed techniques of surface modification. Halloysite is tubule aluminosilicate clay with an external diameter of 50–60 nm, lumen diameter of 12–15 nm and a length of ~1 μm^24^. Its SiO_2_ surface is negative at neutral pH and Al_2_O_3_ inner lumen surface is positive which allows selective loading of clay nanotubes with charged drug molecules^25^ or modification with nanoparticles^26^. Halloysite nanotubes (HNTs) form a stable dispersion in water, their colloidal properties are similar to 100-nm diameter silica nanoparticles. Halloysite has a good biocompatibility, which was assessed for both cell cultures^27^ and whole animals^28^. Various drugs can be loaded in halloysite nanotubes from concentrated solutions or from melt. Dried loaded nanotubes may be kept for a long time, and release drugs while exposed to water within 10–20 hrs (e.g. gentamicin, ciprofloxacin, tetracycline, dexamethasone, and brilliant green). The polymeric coating clogs the tube ends and slows down the drug release rate from hours to days and weeks^29^, allowing for fabrication of drug-delivery systems based on DNA-wrapped nanotubes^25^ and antimicrobial coatings^30^.

Here we report the fabrication of a novel drug delivery system based on HNTs loaded with a model anticancer drug and coated with dextrin (DX) cleavable by intercellular glycosyl hydrolases for controlled release inside cells. As a model drug, we utilised triazole dye brilliant green (BG) capable for suppressing mitochondria in the malignant cells^31^. This platform benefits from the effective uptake of the biocompatible clay nanotubes by human cancer cells, followed by the hydrolysis of the dextrin coating with cellular glycosyl hydrolases^17^, facilitating the release of the drug from the nanotubes and the subsequent inhibition of mitochondria in the cells. The designed cell targeting nanocontainers have to be biocompatible, provide an effective drug encapsulation, and the stimuli-responsive coating should be affected by cytoplasmic factors, preferably enzymes. Halloysite nanotubes served as transmembrane carriers, and utilised two biocompatible compounds–brilliant green and as a cytotoxic substance^31^ and dextrin as an enzyme-activated tube-end stopper^17^.

## Experimental

### Reagents

Ultrapure water was obtained using a Millipore MilliQ reserve osmosis system. Dextrin, brilliant green (BG), 4,6-diamidino-2-phenylindole (DAPI), acridine orange, ethidium bromide, resazurin sodium salt were obtained from Sigma-Aldrich, unless noted. Halloysite nanotubes (HNTs) were purchased from Applied Minerals Inc (USA).

### Cell cultivation

A549 - human lung carcinoma epithelial cells; and Hep3b - hepatocellular carcinoma cells were obtained from American Type Culture Collection (ATCC, Rockville, Maryland, USA). Cells were incubated in humidified atmosphere with 5% CO^2^ at 37 °C. Cells were cultivated in Dulbecco modified minimal Eagle’s medium (DMEM) with L-glutamine supplemented with 100 U mL^−1^ penicillin, 100 μg mL^−1^ streptomycin and 10% of fetal bovine serum (PAA laboratories). Typically, the cells were passaged after approaching 85–90% confluence with 0.05% trypsin-EDTA solution during 5 min incubation and split at a ratio 1:10.

### Fabrication of HNTs loaded with BG and coated by dextrin-stoppers

Prior to loading with BG, HNTs were washed twice with ethanol and then with sterile water and dispersed using an ultrasonic bath^30^. Loading was initiated by adding 1 ml of 1% BG solution in 60% aqueous ethanol was added to 100 mg of washed HNTs, and the solution was sonicated for 30 s. Next, the vial filled with HNTs and BG was placed in vacuum desiccator for 1 h to ensure the vacuum-facilitated suction of BG into the HNTs lumen. Then, to remove the free BG, the suspension was washed twice with sterile water followed by centrifugation. Finally, BG-loaded HNTs were dried at 45 °C and milled to fine powder. The efficiency of BG loading was measured gravimetrically, using the identical samples of HNTs, the control sample (BG free) was used to evaluate the loss of HNTs during washing procedures. 10 mg of BG-loaded HNTs were mixed with 1 ml of aqueous dextrin solution (10 mg mL^−1^) and sonicated for 10 s. Next, the vial containing nanotubes and dextrin was placed in vacuum desiccator for 1 h, followed by washing with sterile water to remove the unbound dextrin. At all stages, the zeta-potential of HNTs in water was monitored using a Malvern Zetasizer Nano ZS instrument with standard U-shaped cells.

### Electron microscopy imaging of HNTs

A Carl Zeiss Libra instrument was used to obtain TEM images of pure and BG-loaded HNTs. A drop of dilute HNTs suspension was placed onto formvar-coated copper grids (Agar) and left to evaporate, then the HNTs were imaged at 120 V accelerating voltage. An Auriga (Carl Zeiss) instrument was used to obtain SEM images of pure and dextrin-coated HNTs (DX-HNTs). Samples were sputter-coated with Au (60%) and Pd (40%) alloy using a Q150R (Quorum Technologies) instrument. Images were obtained at 3 × 10^−4^ Pa working pressure and 10 kV accelerating voltage using the InLens detection mode.

### Release kinetics investigation

The aqueous (10 mg mL^−1^) suspension of BG-loaded HNTs (with and without dextrin stoppers) was incubated while stirring for 24 h. Then the supernatant was removed by centrifugation and analysed at 623 nm using Lambda 35 UV/Vis Spectrophotometer (Perkin Elmer, USA) to estimate the amount of BG released from the HNTs. To estimate the total amount of BG loaded into the nanotubes, which could be released, 100 mg of BG-loaded HNTs in 20 ml water was sonicated for 10 min, then the total amount of the released BG was measured as described above.

### Cellular uptake of DX-HNTs

To investigate the uptake of DX-HNTs, 10^5^ cells were seeded in each well of 12-well culture plates. Then, 25, 50 or 100 μg of DX-HNTs were added into wells. After 24 hours of incubation the plates were analysed using DIC contrast microscopy (Carl Zeiss Axio Observer inverted microscope (Germany) operated using ZEN software) to evaluate the confluent growth of the cells.

### Enhanced dark-field microscopy imaging (EDF)

The observation of distribution of DX-HNTs in cells was performed using EDF microscopy. The cells were grown on glass cover slips and stained with DAPI (1 μg mL^−1^) for 5 min. Then they were fixed by cold acetone (−20 °C) and embedded in the mounting media. EDF microscopy images were obtained using a CytoViva® enhanced dark-field condenser attached to an Olympus BX51 upright microscope equipped with fluorite 100x objectives and DAGE CCD camera. CytoViva® Dual Mode Fluorescence system (UV excitation) was used to visualise DX-HNTs in human cells with DAPI nuclei stain.

### Transmission electron microscopy

TEM images of the thin-sectioned cells and DX-HNTs were obtained using a JEOL 1200 EX microscope operating at 80 kV. The cells were fixed with 2.5% glutaraldehyde, gradually dehydrated using a series of ethanol solutions, embedded into Epon resin, and then thin sections were cut using a LKB ultramicrotome equipped with a diamond knife and mounted on copper grids. The thin-sectioned cells were stained with 2% aqueous uranyl acetate and lead citrate.

### Atomic force microscopy (AFM)

Distribution of DX-HNTs inside cells was investigated using a Dimension Icon microscope (Bruker, USA) using Scan Asyst Peak Force Tapping (in air) mode. Cells were grown on glass cover slips, fixated, washed with water to remove salt and debris, dehydrated and imaged in air. Scan Asyst in Air cantilevers (tip radius −2 nm, spring constant −0.4 n m^−1^) (Bruker) were used throughout. AFM images obtained were processed using Nanoscope Analysis software v1.5 (Bruker, USA).

### Cell viability investigation via viability staining

Fluorescent microscopy was employed to assess the ratios of viable and necrotic cells stained using acridine orange/ethidium bromide (AO/EtBr) dyes. AO intercalates with the DNA and RNA of cells (green fluorescence), whereas EtBr penetrates only into necrotic (dead) cells nuclei (red “dead” fluorescence). Cells were treated with DX-HNTs and then plated into 96-well plates (10^4^ cells per well) and incubated for 24 h. Then 10 μl of AO/EtBr (1% and 0,5% respectively) solution was added to each well for 5 min, then washed twice with DPBS and imaged.

### Cytoskeleton (F-actin) staining

Coverslips with cell monolayer were washed twice with prewarmed DPBS and fixated with 4% paraformaldehyde solution in DPBS for 10 min. Then cells were washed twice with DPBS and permeabilized with 1% Triton X100 for 20 min, then washed with DPBS and subsequently incubated for 30 minutes at room temperature with 1% bovine serum albumin in DPBS (PBSA). Phalloidine conjugated with Alexa Fluor 488 (Life Technology) (methanol solution) was dissolved at 1:40 in DPBS and 200 μl were added to each coverslip for 30 min at room temperature. Then cells were dried with cold acetone and mounted using Eukitt mount.

### Cell index monitoring

xCELLigence Real-Time Cell Analyzer (ACEA Biosciences, USA) was employed to investigate the cell index (a dimensionless parameter which indicates the adhesive properties of cells and proliferation rate by measuring the electrical impedance) in cell cultures studied. Cells were treated with DX-HNTs and then plated in 12-well plate (E-plate) with gold electrodes on bottom at a density 7 × 10^3^ cells per well. The plates were installed in xCELLigence analyser, which was placed in humidified atmosphere with 5% CO^2^ at 37 °C for 24 h. The cell index was monitored in real time using the xCELLigence software.

### LD50 estimation

To estimate the LD50 value for BG-loaded HNTs with and without dextrin coating we used resazurin assay. Cells treated with HNTs were seeded in 96-plates at a density of 7 × 10^3^ cells per well and cultivated for 24 h (the concentration of BG-loaded HNTs with and without dextrin coating was 25, 50 and 100 μg respectively per 10^5^ cells). After 24 hours of incubation 1 μl of resazurin solution (0.4 mg mL^−1^) was added in each well and plates were incubated overnight. The absorbance was measuredat 570 nm using a microplate reader (Multiskan FC, Thermo Fisher Scientific, USA). The efficiency of resazurin reduction by cells is directly proportional to the number of viable cells^32^. The meanings of absorbance of control sample were considered as 100% viability, all further calculations have been done related the number of contact cells. The concentration of BG-loaded HNTs, which caused approximately 50% of cells, was labelled as LD50.

## Results and discussion

**Figure 1:**
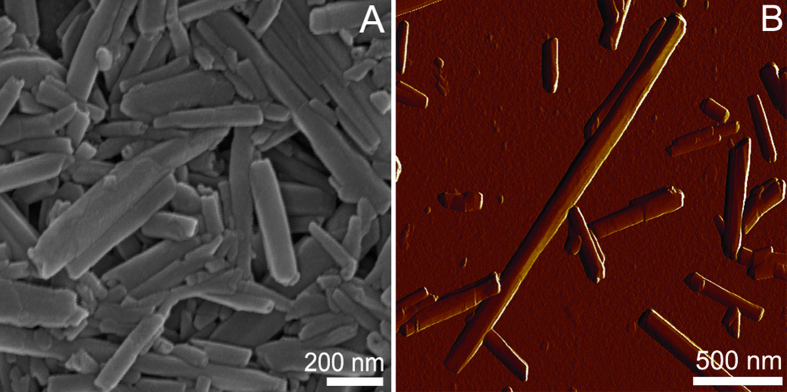
Scanning electron microscopy (**A**) and atomic force microscopy (**B**) images of pristine HNTs.

Halloysite nanotubes employed in this study were characterised using scanning electron and atomic force microscopy (Fig. 1), to demonstrate the typical sizes of the HNTs (ca. 15 nm lumen and 50 nm outer diameter, and 1–1.5 μm length).

**Figure 2:**
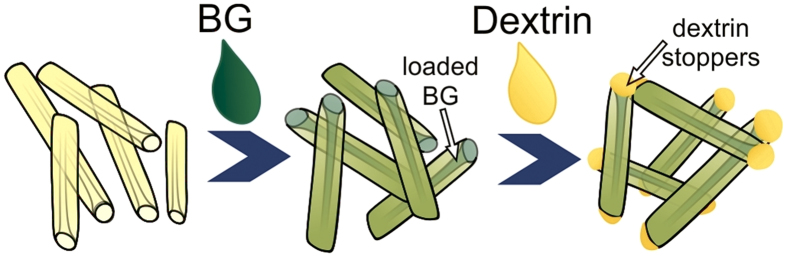
Fabrication of brilliant green-loaded HNTs and the subsequent coating with dextrin stoppers.

Our strategy is schematically shown in (Fig. 2). HNTs lumens were first loaded with brilliant green using the straightforward vacuum suction technique^30^. Next, BG-loaded nanotubes were directly surface-functionalised with dextrin, which produced an enzyme-responsive coating with tube-end clogging to ensure content of BG molecules during delivery and induced release inside the cells.

**Figure 3:**
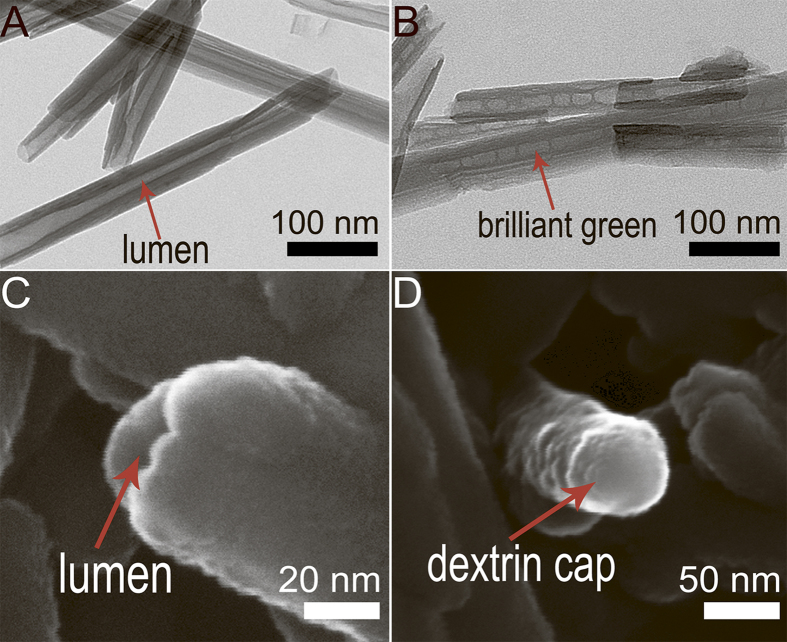
(**A**)—TEM image of pristine HNTs; (**B**)—TEM image of brilliant green loaded halloysite, BG is visible inside the lumen; (**C**)—SEM image of an end of pristine nanotube with open lumen; (**D**)—SEM image of a dextrin cap on the end of the functionalised nanotube.

Transmission electron microscopy (TEM) images (Fig. 3 A,B) demonstrate the brilliant green loading into the lumens of halloysite after 1 h incubation with 1 wt% BG in ethanol, followed by washing, drying, and milling to fine powder, as described in Experimental section (Figure S1, Supporting Information).

The loading efficiency (40 mg per 100 mg HNTs) was determined gravimetrically. This exceeds the halloysite nanotube lumen volume and indicates that the loading occurs both inside the tubes and in the outside pores formed by loosely rolled external alumosilicate sheet.

**Figure 4:**
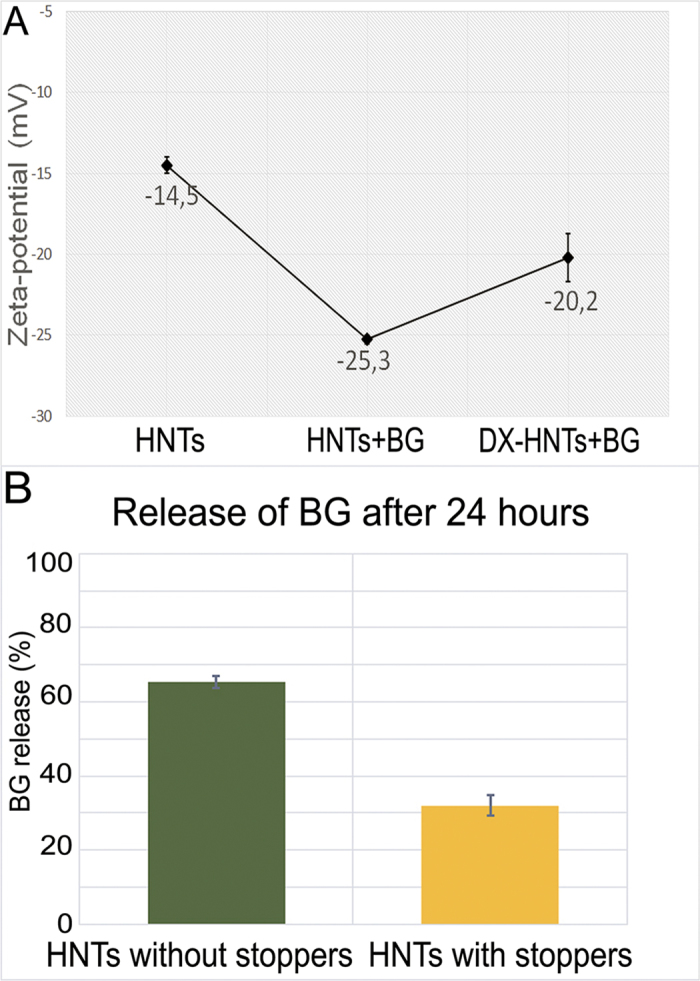
(**A**)—Zeta-potential of HNTs after loading of brilliant green and consecutive coating with dextrin; (**B**)—release of brilliant green from dextrin-coated and dextrin-free nanotubes after 24 hrs in water.

Next, the BG-loaded nanotubes were similarly coated with dextrin via vacuum-facilitated deposition, resulting in formation of physically-adsorbed dextrin layer on HNTs (DX-HNTs), which could be clearly seen in scanning electron microscopy (SEM) images (Fig. 3 C,D). Zeta-potential of HNTs (Fig. 4 A) was monitored after BG loading and dextrin coating, suggesting that dextrin coating will reduce the release. In fact, 60 wt% release of BG from the uncapped halloysite took 24 hrs. Dextrin stoppers reduce the release of BG twofold, as compared with uncoated tubes, allowing for enhanced drug delivery with tubes opening (Fig. 4 B).

This technique of enzyme-activated carriers with polysaccharides stoppers is easier to produce since it does not require any covalent modification of nanotubes, unlike in the earlier reported system based on chemical binding of saccharides onto mesoporous nanoparticles^17^.

**Figure 5:**
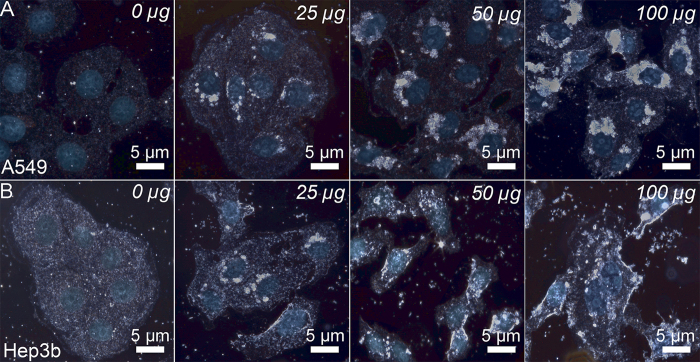
Enhanced dark-field and fluorescence (nuclei are stained with DAPI) images of A549 (**A**) and HEP3b (**B**) cells incubated with increasing concentrations of DX-HNTs per 100 000 cells (indicated on the images).

The important issue in using halloysite nanotubes as drug delivery vehicles is the ability of HNTs to penetrate through the cellular membranes. It appears that the efficiency of HNTs uptake depends on the cells growth rate of different cell cultures. We have chosen two types of cells—adenocarcinomic human alveolar basal epithelial cells (А549) and human hepatoma cells (Hep3b). These cells exhibit the significantly different proliferation rates, which allows to investigate the uptake as a function of the cell growth speed. These cells were subjected in culture with increasing concentrations of halloysite formulation of DX-HNTs (from 25 to 100 μg per 100 000 cells, BG-free). After 24 hours of incubation, cells were microscopically analysed. First, we employed the enhanced dark-field (EDF) microscopy (Fig. 5), demonstrating that the uptake behaviour of A549 cells and Hep3b cells was distinctly different. Lung carcinoma cells internalised DX-HNTs and concentrated them in perinuclear regions (counter-stained with DAPI and visualised using transmission-light fluorescence microscopy) as clearly visible aggregates, whereas Hep3b cells appear to randomly distribute the DX-HNTs in cytoplasm.

**Figure 6:**
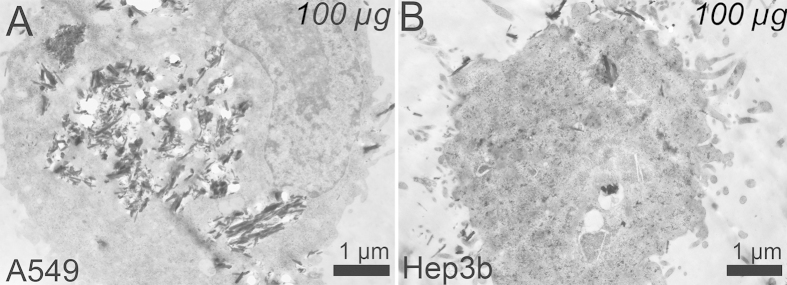
TEM images of A549 (**A**) and HEP3b (**B**) cells incubated with 100 μg DX-HNTs per 100000 cells).

Suspended cells, detached from the culture plates, were imaged to demonstrate the spatial distribution of DX-HNTs inside these two cultures (Figure S2, Supporting Information), demonstrating the differential uptake. These findings have been confirmed further using TEM images (Fig. 6) of A549 and Hep3b cells incubated with 100 μg DX-HNTs per 100 000 cells, the highest concentration used. TEM images suggest that in A549 cells nanotube aggregates seen in dark-field images (Fig. 5 A) are randomly distributed in lysosomes, which could facilitate the enzymatic decomposition of dextrin tube-end stoppers with subsequent enhanced cargo release.

**Figure 7:**
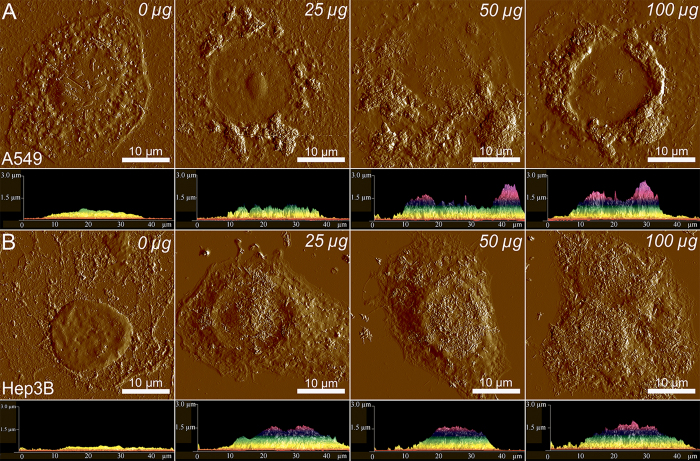
AFM images and side profiles of A549 (upper row) and Hep3b (lower row) cells treated with increasing concentrations of DX-HNTs (indicated on each image).

On the contrary, Hep3b cells accumulate nanotubes preferentially on cellular membranes, thus reducing the access of intracellular glycosyl hydrolases (Fig. 5 B). We also assume that in case of Hep3b cells nanotubes remain suspended in media rather than being actively internalised by cells. We employed atomic force microscopy (AFM) to investigate the distribution of DX-HNTs in fixated dry samples of A549 and Hep3b cells (Fig. 7). Typically, AFM imaging demonstrates surface-absorbed nanotubes, however, as the imaging is applied to dried samples where the volume of the cells is no longer preserved, cell membranes collapse and solid nanotubes aggregates can be probed using AFM. One can clearly distinguish relatively large amorphous and quite smooth aggregates inside the A549 cells from well-resolved surface-adsorbed nanotubes on Hep3b cells membranes (numerous single tubes can be seen). We attribute the smooth topography of larger DX-HNTs aggregates in A549 cells to the dried membrane\cytoplasm films over the internalized nanotube aggregates. AFM images confirm that the overall morphology of both A549 and Hep3b cells remains unaffected by the increasing concentrations of DX-HNTs. However, we noted that internalized DX-HNTs in A549 formed crater-like concentric circular regions around the nucleus (seen both in top-view and side-view images), while only negligible numbers of nanotubes were detected in the distal regions or on the membrane close to the nucleus. On the contrary, in Hep3B cells the nanotubes the detected mostly on the cellular membranes contributing to the increased height of the cupola-like position of the cells (side-view). These results suggest that the DX-HNTs are preferentially internalised by certain types of cells, which may be exploited in differential treatment of the target cells. Previously, several reports indicated that the HNTs are relatively non-toxic towards human cells^27^, microorganisms^33^ and soil organisms^28^. Here we carefully investigated the effects of dextrin-coated HNTs on A549 and Hep3b cells employing a set of physiological activity test. First, we stained the cells treated with the increasing concentrations of DX-HNTs with acridine orange\ethidium bromide dyes, allowing distinguishing the viable cells from the necrotic ones.

**Figure 8:**
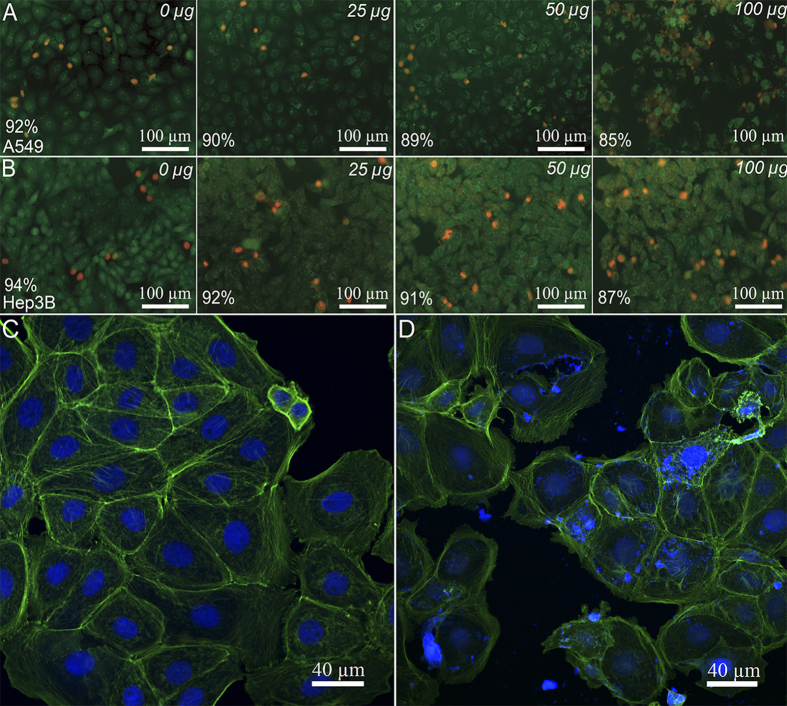
Viability of human cells treated with the increasing concentrations of DX-HNTs. Fluorescence microscopy images of viable A549 (**A**) and Hep3b (**B**) cells (viable cell count is indicated on each image); F-actin filaments stained in intact (**C**) and DX-HNTs-treated A 549 cells.

Viable cells appear green on fluorescence microscopy images, suggesting the intact membranes, while the lack of red ethidium bromide-mediated fluorescence in viable cells confirms the integrity of cellular membranes (unlike in necrotic cells). The results (Fig. 8) demonstrate that DX-HNTs per se, even at higher concentrations, do not affect the both A549 and Hep3b cells, since the percentage of viable cells is only slightly reduced.

In addition, we investigated the effects of DX-HNTs on cytoskeleton formation. We found that the HNTs taken up by A549 (Fig. 8C and D) and Hep3b (data not shown) cells do not induce any detectable changes in cytoskeleton organisation in cells.

**Figure 9:**
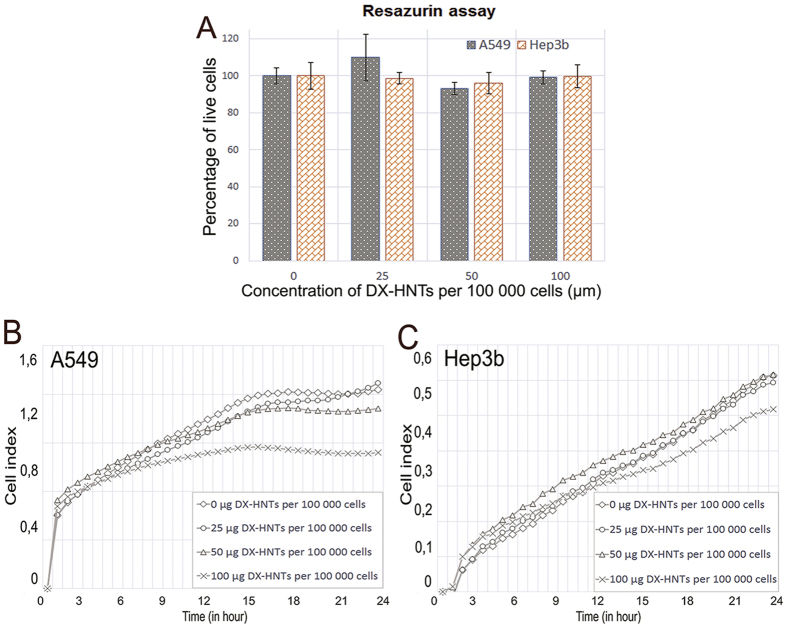
(**A**)—resazurin transformation assay results; (**B**, **C**)—cell index curves of A549 (**B**) and Hep3b (**C**) cells demonstrating the unaffected attachment and growth rates.

Next, we analysed the redox metabolism indicator resazurin transformation in DX-HNTs-treated cells. Viable cells reduce resazurin into the pink resorufin product detectable using spectrophotometry. We found that metabolic activity in DX-HNTs-treated cells was not affected if compared with control cells (Fig. 9A). We also investigated the attachment and growth rate of DX-HNTs-treated cells employing the real-time Cell Index monitoring. As shown in Fig. 9 B, C, the higher (100 μg DX-HNTs per 10^5^ cells) concentration of nanotubes did somewhat reduce the growth dynamics, however the lower concentrations investigated did not affect the cells. Hence, we concluded that HNTs equipped with dextrin stoppers are not toxic to the cells.

**Figure 10:**
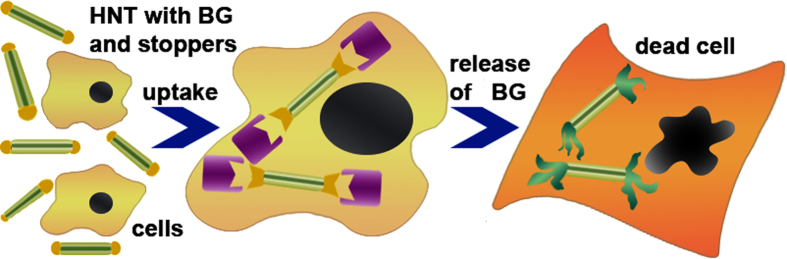
A scheme of the enzyme-activated anticancer drug delivery system based on drug-loaded HNTs with dextrin stoppers.

Finally, we focused on the delivery of brilliant green into A549 and Hep3b cells. We assume that the dextrin coating will seal the drug inside the nanotubes before their internalisation, while after, the coating is hydrolysed by intercellular glycosyl hydrolases, the nanotube content will be released into the cytoplasm and kill the target cell (Fig. 10).

**Figure 11:**
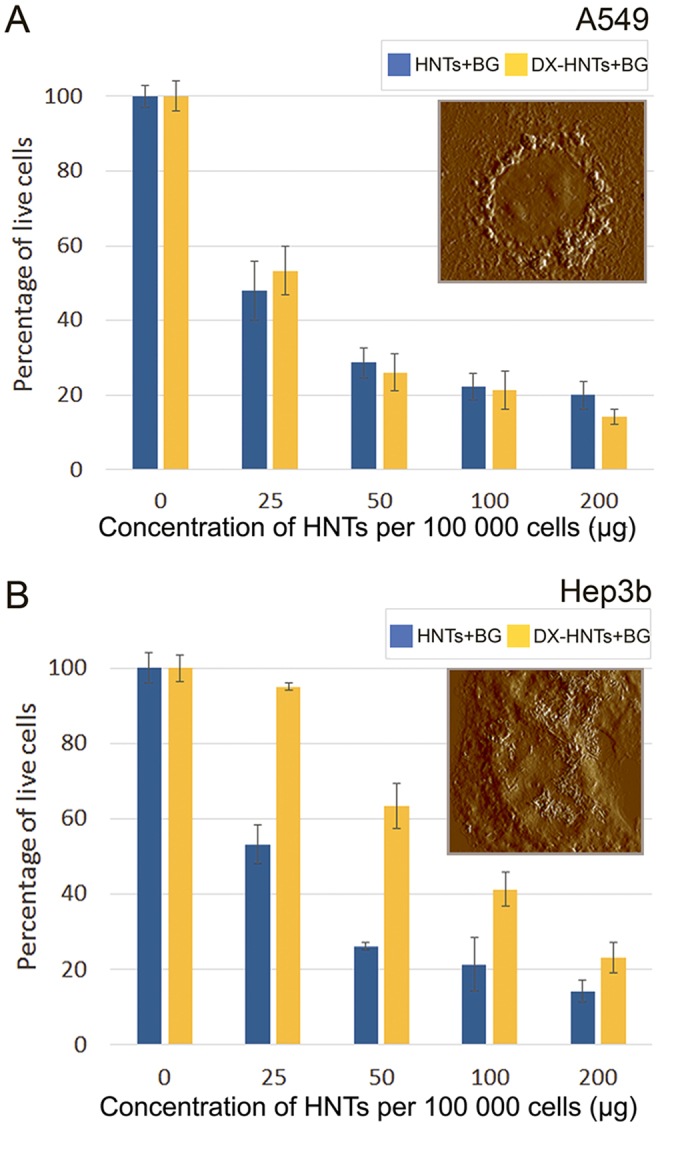
Resazurin assay results demonstrating the LD50 value (50% death level) of BG-loaded HNTs for A549 and Hep3b cells. The two-fold increase of LD 50 for Hep3b cells occurs due to the reduced uptake of HNTs if compared with A549 cells. Insets show AFM images of distribution of DX-HNTs in cells.

We found that the dextrin stoppers substantially reduce the toxicity of the nanotube BG formulation towards Hep3b (two-fold reduction) (Fig. 11 A, Figure S3) whereas the median lethal dose (LD50) value of BG nanotube formulation for A549 cells did not depend on dextrin coating (Fig. 11 B, Figure S4). This may be related to the differential uptake of DX-HNTs in these two types of human cancer cells discussed above. The A549 cells take up most of the DX-HNTs nanocarriers available from the media, on the contrary, the uptake in Hep3b cells is smaller (Figs 6 and 7), and BG-loaded DX-HNTs do not have significant influence on Hep3b cells even after decomposition of enzyme-activated coating. Most of the BG-loaded DX-HNTs remain outside the cells and dextrin stoppers are not affected by the lysosome enzymes (no release of brilliant green occurred). The reduced amount of free brilliant green spontaneously released from the nanotubes is not sufficient to kill the Hep3b cell, and they remain viable and continue to proliferate (Figure S4). As a result, the selective killing of the A549 cells can be achieved, while Hep3b cells remain viable at the same concentration (i.e., 25–50 μg per 10^5^ cells in case of BG-HNTs).

## Conclusions

We developed a novel strategy for drug loading into clay nanotubes coated with dextrin to clog the tube opening until the cell absorbs these nanocarriers. The accumulation and enzymatically induced release of drug occurred exclusively in cells prone to internalization of the nanotubes with higher proliferation rates, which is a characteristic of malignant cells. It means that non-malignant cells will not suffer from the introduction of halloysite with anticancer drug, as a result, drug-loaded DX-HNTs will be accumulated selectively in tumor cells. This would allow using several weak drugs with low cytotoxic effects, when the high concentration of agent inside cells is required to damage and kill them, whereas at lower concentrations these drugs are not harmful. The non-cancerous cells with bad “HNTs-appetite” resulting from the slow proliferation and level of metabolism will not be affected by the drug, unlike current directly-applied anticancer drugs.

## Author Contributions

R.F.F. and Y.M.L. conceived the idea and designed the experiments; M.R.D., E.A.N., and R.F.F. performed the experiments, analyzed the data and prepared the figures; M.R.D., R.F.F. and Y.M.L. interpreted the results and wrote the manuscript. All authors reviewed the manuscript.

## Additional Information

**How to cite this article**: Dzamukova, M. R. *et al.* Enzyme-activated intracellular drug delivery with tubule clay nanoformulation. *Sci. Rep.* **5**, 10560; doi: 10.1038/srep10560 (2015).

## Supplementary Material

Supporting Information
