# Supplementary material for: Enzyme-activated intracellular drug delivery with tubule clay nanoformulation
Source: Sci Rep. 2015 May 15;5:10560. doi: 10.1038/srep10560 (PMC4432568; doi:10.1038/srep10560)
Supplement: Supporting Information [file srep10560-s1.doc]

*Supporting information*

Enzyme-activated intracellular drug delivery with tubule clay nanoformulation

M. R. Dzamukova,^a^ E. A. Naumenko^a^, A. R. Badrutdinov,^a^ Y. M. Lvov,^b^ R. F. Fakhrullin^a^*

1 - Bionanotechnology Lab, Department of Microbiology, Institute of Fundamental Medicine and Biology, Kazan Federal University, Kreml uramı 18, Kazan, Republic of Tatarstan, Russian Federation, 420008, [kazanbio@gmail.com](mailto:kazanbio@gmail.com)

2 - Institute for Micromanufacturing, Louisiana Tech University, 911 Hergot Ave., Ruston, LA, USA, 71272

* Corresponding author, Rawil F. Fakhrullin, kazanbio@gmail.com


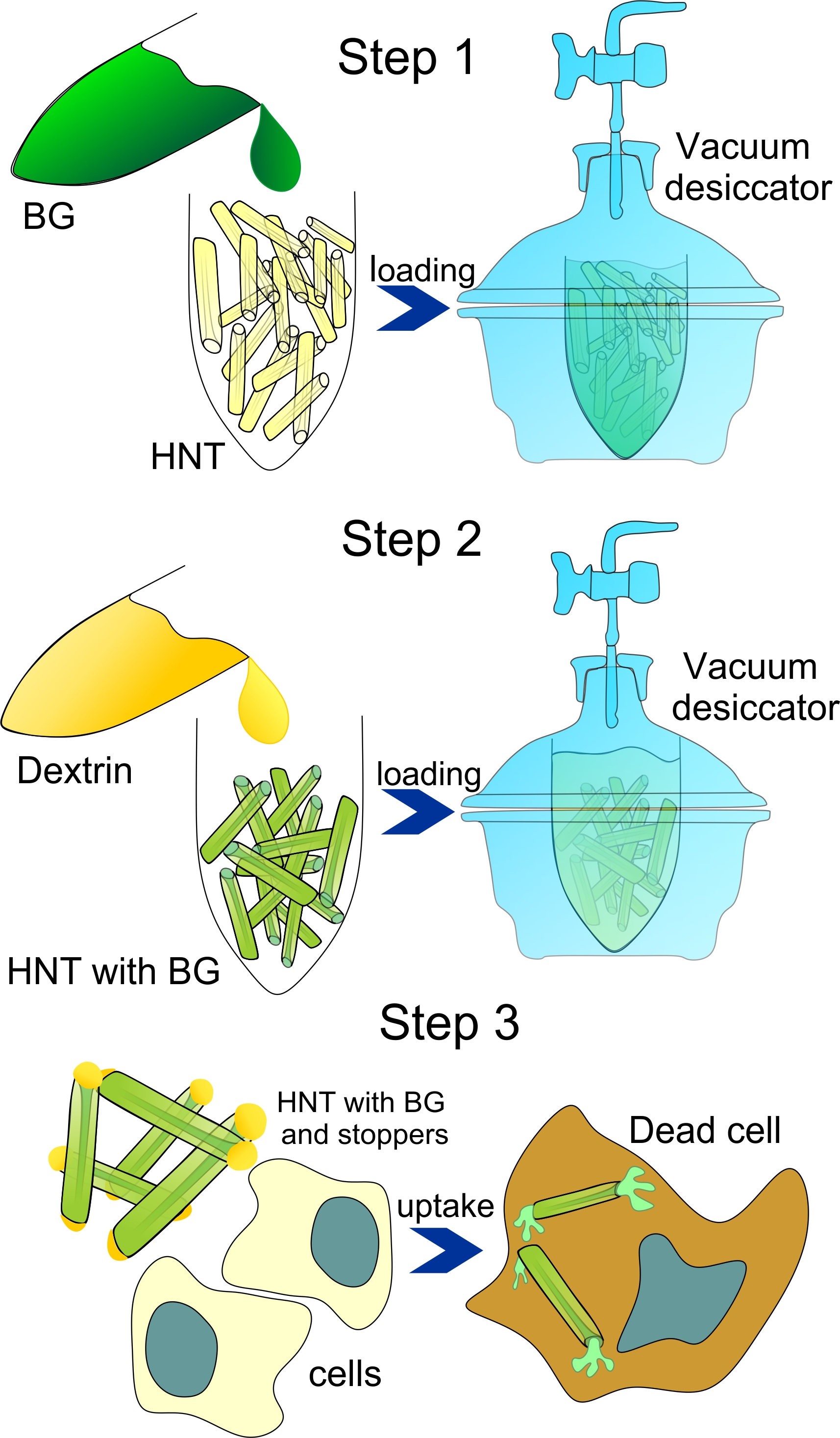


**Fig. S1** A sketch illustrating the preparation of BG-loaded HNTы with enzyme-activated dextrin coating (step 1 and 2), and their further application as a targeted drug delivery system with anticancer activity (step 3).


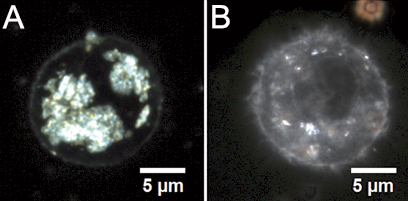


**Fig. S2** Enhanced dark-field microscopy images of HNTs in suspended A549 (a) and Hep3b (b) cells (seen as bright light-scattering spots inside the cells, for real-time imaging see Videos 1 and 2).


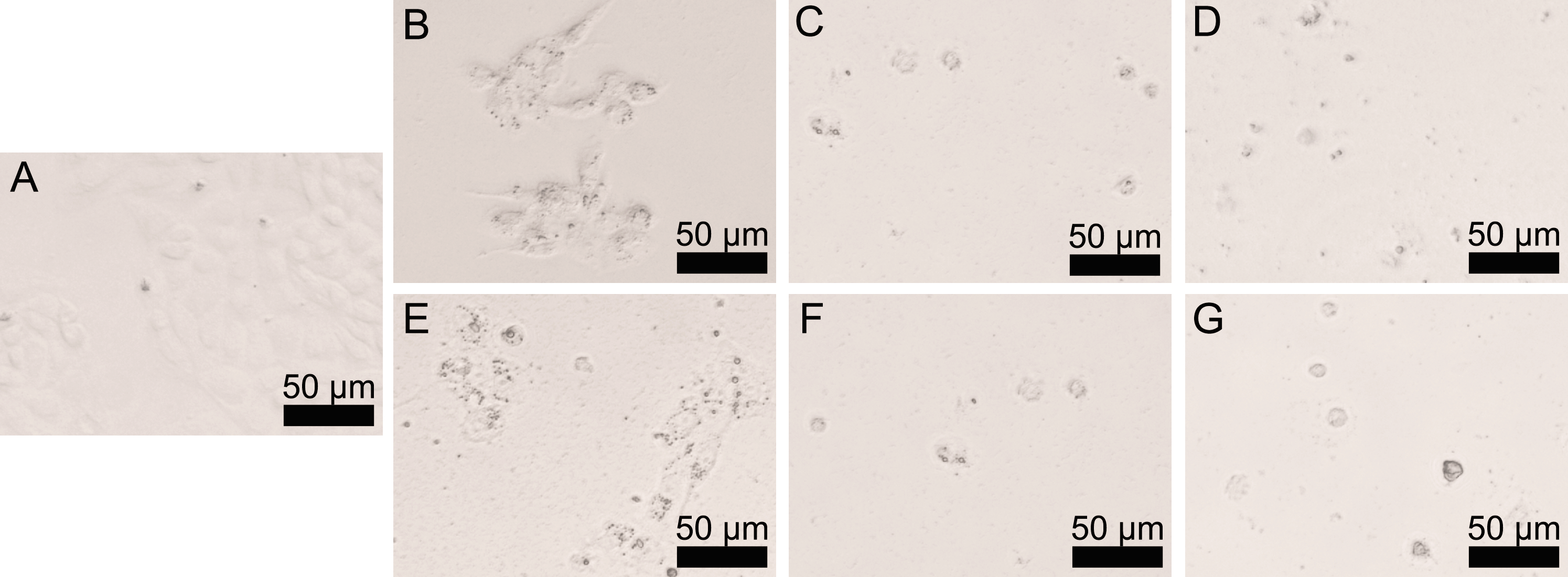


**Fig. S3** The bright-field microscopy images of A549 cells threated with the increasing concentrations of HNTs loaded by BG without dextrin coating (B-D) and coated with dextrin (E-G) after 24h of incubation: HNTs-free (control) (A); 25 µg of HNTs per 10^5^ cells (B, E); 50 µg of HNTs per 10^5^ cells (C, F); 100 µg of HNTs per 10^5^ cells (D, G).


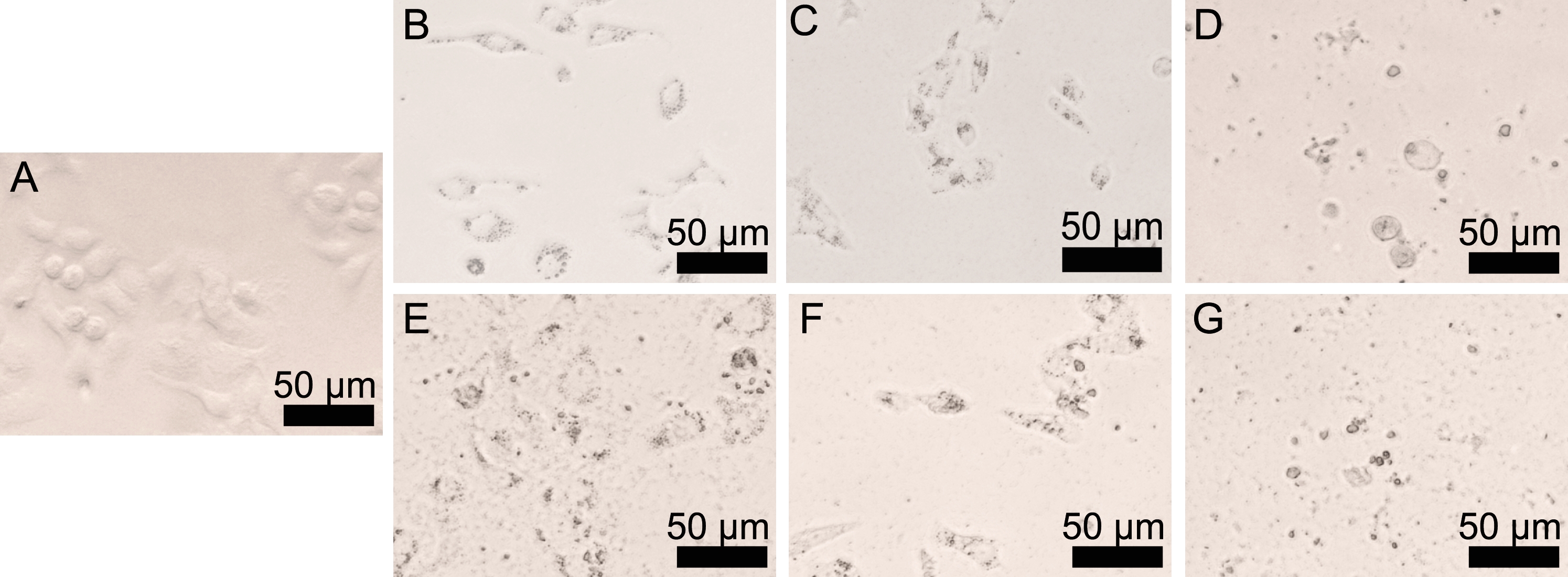


**Fig. S4** The bright-field microscopy images of Hep3b cells threated with the increasing concentrations of HNTs loaded by BG without dextrin coating (B-D) and coated with dextrin (E-G) after 24h of incubation: HNTs-free (control) (A); 25 µg of HNTs per 10^5^ cells (B, E); 50 µg of HNTs per 10^5^ cells (C, F); 100 µg of HNTs per 10^5^ cells (D, G).
